# Association of systemic inflammatory indices with anthropometric measures, metabolic factors, and liver function in non-alcoholic fatty liver disease

**DOI:** 10.1038/s41598-024-63381-5

**Published:** 2024-06-04

**Authors:** Sara Arefhosseini, Taha Aghajani, Helda Tutunchi, Mehrangiz Ebrahimi-Mameghani

**Affiliations:** 1 Student Research Committee, Tabriz University of Medical Sciences, Tabriz, Iran; 2 Department of Animal Biology, Faculty of Natural Sciences, University of Tabriz, Tabriz, Iran; 3 Endocrine Research Center, Tabriz University of Medical Sciences, Tabriz, Iran; 4 Nutrition Research Center, Department of Biochemistry and Diet Therapy, Faculty of Nutrition & Food Sciences, Tabriz University of Medical Sciences, Tabriz, Iran

**Keywords:** Inflammation, Systemic inflammatory indices, Metabolic factors, Liver function, Non-alcoholic fatty liver disease, Non-alcoholic fatty liver disease, Dyslipidaemias, Metabolic syndrome, Obesity

## Abstract

The present cross-sectional study aimed to explore the relationship between systemic inflammatory indices (SIIs) and anthropometric measures, metabolic, and liver function biomarkers in patients with non-alcoholic fatty liver disease (NAFLD). This study was carried out on 238 NAFLD patients with overweight or obesity, aged 18–55 years. Anthropometric measurements were done and body mass index (BMI), waist-to-hip ratio (WHR), and waist-to-height ratio (WHtR) were estimated. Metabolic factors including serum glucose, lipid profile, liver function biomarkers, and complete blood cell count were assessed after a 24-h fasting state. SIIs including the ratios of neutrophil to lymphocyte (NLR), monocytes to lymphocyte (MLR), platelet to lymphocyte (PLR), and monocytes to high-density lipoprotein cholesterol (MHR) were calculated. Results indicate that apart from PLR, all of the SIIs significantly changed by increasing steatosis severity (all *p* < 0.05). Moreover, changes in NLR showed a significant association with anthropometric indices including waist circumference (*p* = 0.032), BMI (*p* = 0.047), and WHtR (*p* = 0.002), as well as levels of fasting blood sugar (*p* = 0.045), triglycerides, (*p* = 0.025) and low-density lipoprotein cholesterol (*p* = 0.006). The findings also indicate the relations between lipid profile and all studied SIIs, notably MHR and MLR. All of the SIIs exhibited associations with some liver function indices as well. MHR was positively correlated with the metabolic risk factors of NAFLD while, oppositely, PLR was considered as a preventive marker of NAFLD.

## Introduction

Chronic low-grade inflammation is considered as a main driver of liver-related metabolic disturbances^1^. Both systematic intra- and extra-hepatic inflammatory consequences trigger the progression of a cluster of pathological entities including hepatic steatosis, insulin resistance, oxidative stress, and gut dysbiosis, and consequently, emerged as non-alcoholic fatty liver disease (NAFLD)^2,3^. In 2020, metabolic dysfunction-associated fatty liver disease (MAFLD) was proposed as a more appropriate term than NAFLD, because this nomenclature better defines the pathophysiology of the disease and its related metabolic disorders. Chronic inflammation and fibrosis are considered to be the main factors in the pathogenesis of MAFLD^4^.

NAFLD, the most common hepatic disease, is the cornerstone of a plethora of metabolic disorders including type 2 diabetes mellitus (T2DM), metabolic syndrome (Mets), and cardiovascular disease (CVD) that are inter-related through systemic inflammation^3^. Accordingly, the sustained activation of pro-inflammatory signaling pathways accelerates NAFLD progression to steatohepatitis, liver fibrosis, and even, carcinoma^1^.

Complete blood count (CBC) compartments including platelets, lymphocytes, monocytes, and neutrophils as prominent immune cells, simultaneously mediate inflammatory and metabolic processes^2,5^. Monocytes, as a part of the phagocytic immune system, exert critical functions in inflammation, while platelets and neutrophils are involved in cytokines and chemokines production^2,6^. Systemic inflammatory indices (SIIs) including monocyte-to-lymphocyte ratio (MLR), neutrophil-to-lymphocyte ratio (NLR), platelet-to-lymphocyte ratio (PLR), and monocyte-to-high-density lipoprotein cholesterol (HDL-C) ratio (MHR) have recently achieved great attention in clinical settings^6^. Notwithstanding the feasibility of these blood routine tests, SIIs are simple, non-invasive, and useful markers of the prognosis, diagnosis, and risk assessment for metabolic conditions^2,6,7^. In the case of NAFLD, due to problems in their sensitivity, validation, and expensiveness, SIIs could be preferred to current screening and diagnostic tools such as biopsy, imaging techniques, and even liver-related indices (e.g. fatty liver index, NAFLD fibrosis score, and hepatic steatosis index)^8,9^. The current body of evidence suggests the relation between SIIs, especially MHR, and NLR, with T2DM, Mets, and NAFLD outcomes^2,10,11^. Indeed, PLR has been even suggested as a protective factor of NAFLD^12^. To our knowledge, there is limited research on correlations between SIIs and NAFLD metabolic outcomes as well as the severity^8^. Therefore, the current cross-sectional study aimed to investigate the relationship between SIIs and anthropometric indices, metabolic and liver function biomarkers in patients with NAFLD.

## Materials and methods

### Study design

The present cross-sectional study was done on 238 NAFLD patients with overweight or obesity (body mass index (BMI) ≥ 25 kg/m^2^) (both genders and ages ranged from 18 to 55 years). The diagnosis of NAFLD was done by ultrasonography based on Hamaguchi et al.^13^ definition. The patients who were pregnant, lactating, menopause, athletes, bariatric surgery, suffering from other chronic diseases and malignancies, receiving antihypertensive, anti-hyperlipidemic, and anti-diabetic medications as well as oral contraceptives, nutritional supplements three months before the study were excluded. The study protocol was approved by the Ethics Committee of Tabriz University of Medical Science (IR.TBZMED.REC.1401.689). Moreover, informed consent was fulfilled for each subject after considering the study criteria and full explanation regarding study objectives and protocol.

### Sample size

The sample size was estimated as 238 according to the prevalence rate of NAFLD in Iran, reported by Moghaddasi far et al.^14^ and by considering a 95% confidence level and 6% error using G- power software (Heinrich-Heine-Universität Düsseldorf, Düsseldorf, Germany). The calculation resulted in a total of 231 sample size. Considering the possiblity of nonrespodents during the data collection process, the number of participants was increased by 15%, leading to a total of 264 participants.

### Data collection

Personal details including demographic and socioeconomic status as well as behavioral details e.g. smoking, alcohol drinking, medical history, and taking supplements were obtained through a face-to-face interview by a trained nutritionist. Weight and height were measured in fasting state using a calibrated stadiometer (Seca, Hamburg, Germany) with light cloths and no shoes to the nearest 100 g and 5 mm, respectively. Waist circumference (WC) was measured at the midpoint between the lower border of the rib cage and iliac crest with 0.1 cm precision whereas hip circumference (HC) was assessed around the widest part of the buttocks to the nearest 0.1 cm using a non-stretchable anthropometric tape. Then, anthropometric indices including BMI, WC to HC ratio (WHR), and WC to height ratio (WHtR) were estimated.

### Laboratory assay

After 12–14 h of overnight fasting, 10 ml blood samples were collected and divided into two tubes; 5 ml whole blood in EDTA-containing vacuum blood collection tubes (Vacutainer K2E) and 5 ml vacuumed gel separator tubes to be centrifuged for sera and then stored at -80 ^◦C^ until the end of the study. Automated and daily-calibrated Coulter CBCH1 counter was used for assessing total white blood cell count, neutrophil, lymphocyte, monocyte count per mm3 as well as platelets count in whole blood and then, the ratios NLR, MLR, PLR and MHR were calculated as SIIs. In addition, the sera were used to determine metabolic parameters including fasting blood sugar (FBS), triglycerides (TG), total cholesterol (TC), and HDL-C using commercial kits (Pars-Azmoon Co., Tehran, Iran), and an auto-analyzer (Hitachi 902, Tokyo, Japan) as well as alanine aminotransferase (ALT) and aspartate aminotransferase (AST) based on the International Federation of Clinical Chemistry procedure to calculate AST to ALT ratio (AST/ALT)^15^. Electrochemiluminescence immunoassay was applied to determine serum ferritin levels. Then, low-density lipoprotein cholesterol (LDL-C) was estimated using Friedewald formula as follows^16^:

$$ {\text{LDL - C}}\;\left( {{\text{mg}}/{\text{dL}}} \right) = {\text{TC}}\;\left( {{\text{mg}}/{\text{dL}}} \right) - {\text{HDL - C }}\left( {{\text{mg}}/{\text{dL}}} \right) - {\text{TG}}\;\left( {{\text{mg}}/{\text{dL}}} \right)/5. $$

### Statistical analysis

Data was analyzed using Statistical Package for Social Analysis (SPSS Inc., ver. 20, Chicago, IL, USA). The Kolmogorov–Smirnov test was used to check the normality distribution of continuous variables whereas Chi-square test was used for the relationship between categorical variables. Differences in symmetric and asymmetric continuous variables were done using two-independent sample t- and Mann–Whitney U-tests between the two groups, respectively. For comparing the mean of the studied variables in more than two groups, analysis of variance was applied. The association between continuous variables was checked using Pearson and Spearman correlation coefficient for symmetric and asymmetric data, respectively. *p* < 0.05 was considered as statistically significant.

### Ethics approval and consent to participate

All procedures performed in this study were in accordance with the ethical standards of the Ethics Committee of Tabriz University of Medical Science (IR.TBZMED.REC.1401.689) and all eligible patients signed an informed written consent form.

## Results

Table 1 shows participants' characteristics. More than half of the participants were female, married, and with university degrees. Overweight and mild steatosis were found in 71% and 58.8% of the studied patients, respectively. Most of the patients showed central obesity in terms of WC and WHtR, while around one-third of them had high WHR.

**Table 1:** Participants’ charachteristics.

	Total N = 238
	N (%)
Sex	
Male	105 (44.1)
Female	133 (55.9)
Marital status	
Single	41 (17.2)
Married	197 (82.8)
Education	
Less than diploma	75 (31.5)
Diploma	23 (9.7)
University degree	140 (58.8)
Liver steatosis	
Mild (grade I)	140 (58.8)
Moderate (Grade II)	98 (41.2)
BMI status (Kg/m^2^)	
Overweight (25–29.9)	169 (71.0)
Obese (30–39.9)	69 (29.0)
High WC^†^	234 (98.32)
High WHR^††^	90 (37.82)
High WHtR^†††^	238 (100)

	Mean (SD)
Age (years)	37.94 (8.36)
Weight (Kg)	91.43 (16.24)
Height (m)	1.67 (0.10)
WC (cm)	106.59 (10.32)
HC (cm)	114.38 (8.62)
BMI (Kg/m^2^)	32.58 (4.47)
WHR	0.93 (0.06)

*BMI* body mass index, *WC* waist circumference, *HC* hip circumference, *WHR* waist to hip ratio, *WHtR* waist-to-height ratio.

^†^Values > 88 cm in females and > 102 cm in males.

^††^Values > 0.90 in females and > 0.95 in males.

^†††^Values > 0.50 in both genders.

Between-gender comparisons in the studied parameters are shown in Table 2. There were significant differences in all anthropometric measures and indices between the genders, i.e. mean WC and WHR were significantly greater in males than females while mean BMI and WHtR were higher in women than men (*p* < 0.05). Among metabolic factors, significant between-gender differences in serum levels of HDL-C, AST, ALT, AST/ALT, and ferritin were found (*p* < 0.001). Mean platelets and monocytes were statistically different between genders (*p* < 0.001 and *p* = 0.012, respectively). Although MHR in males was significantly greater than females (*p* < 0.001), MLR and PLR were higher in women than men (*p* = 0.047 and *p* = 0.005, respectively).

**Table 2:** Participants’ characteristics by gender.

	Total N = 238 Mean (SD)	Males N = 105 Mean (SD)	Females N = 133 Mean (SD)	*p*
Age (yrs.)	37.94 (8.36)	38.29 (8.60)	37.75 (8.10)	0.624*
Weight (Kg)	91.43 (16.24)	98.28 (17.62)	86.30 (12.68)	< 0.001*
Height (m)	1.67 (0.10)	1.76 (0.07)	1.60 (0.06)	< 0.001*
WC (cm)	106.59 (10.32)	109.13 (10.99)	104.75 (9.17)	0.001*
HC (cm)	114.38 (8.62)	113.16 (8.41)	115.44 (8.57)	0.041*
BMI (Kg/m^2^)	32.58 (4.47)	31.81 (5.10)	33.22 (3.78)	0.015*
WHR	0.93 (0.06)	0.96 (0.05)	0.91 (0.06)	< 0.001*
WHtR	0.64 (0.06)	0.62 (0.06)	0.65 (0.06)	< 0.001*
FBS (mg/dl)	93.98 (10.38)	93.55 (10.52)	94.42 (10.25)	0.522*
TG (mg/dl)	158.16 (78.14)	157.79 (83.36)	159.00 (73.67)	0.906*
TC (mg/dl)	178.63 (34.92)	178.16 (28.71)	179.30 (39.04)	0.794*
HDL-C (mg/dl)	44.44 (9.91)	42.62 (7.91)	46.02 (10.95)	< 0.001*
LDL-C (mg/dl)	136.66 (57.68)	141.40 (57.15)	133.15 (58.25)	0.277*
ALT (U/L)	31.07 (19.57)	38.27 (18.98)	25.54 (18.11)	< 0.001*
AST (U/L)	24.51 (10.13)	27.38 (10.74)	22.30 (9.00)	< 0.001*
AST/ALT	0.92 (0.40)	0.78 (0.26)	1.03 (0.44)	< 0.001*
Ferritin (ng/mL)	90.88 (75.80)	119.61 (90.64)	69.55 (52.40)	< 0.001**
WBC (*10^9^/L)	9.81 (0.22)	10.02 (2.11)	9.69 (2.16)	0.203*
Monocytes (*10^9^/L)	0.61 (0.49)	0.62 (0.24)	0.61 (0.62)	0.012**
Lymphocytes (*10^9^/L)	3.42 (0.73)	3.46 (0.70)	3.39 (0.76)	0.624*
Neutrophils (*10^9^/L)	5.54 (1.37)	5.57 (1.29)	5.51 (1.43)	0.943*
Platelets (*10^9^/L)	250.33 (59.91)	236.90 (65.83)	260.94 (52.72)	< 0.001*
MHR(*10^8^/mmol)	5.83 (5.33)	6.06 (2.79)	5.64 (6.73)	< 0.001**
MLR	0.18 (0.14)	0.18 (0.07)	0.19 (0.17)	0.047**
NLR	1.74 (0.80)	1.69 (0.58)	1.79 (0.94)	0.995**
PLR	77.74 (29.65)	72.07 (26.49)	82.81 (31.30)	0.005*

Mean ± standard deviation (SD) is presented for data.

Bold values indicate statistically significant *p* < 0.05.

*WC* waist circumference, *HC* hip circumference, *BMI* Body mass index, *WHR* waist to hip ratio, *WHtR* waist to height ratio, *FBS* fasting blood sugar, *TG* triglycerides, *TC* total cholesterol, *HDL-C* high-density lipoprotein cholesterol, *LDL-C* low-density lipoprotein cholesterol, ALT alanine aminotransferase, *AST* aspartate aminotransferase, *AST/ALT* AST to ALT ratio, *WBC* white blood cell count, *MHR* monocyte to HDL-C ratio, *MLR* monocyte to lymphocyte ratio, *NLR* neutrophil to lymphocyte ratio, *PLR* platelets to lymphocytes ratio.

*p-value for Two-independent Sample t-test.

**p-value for Mann–Whitney U-test.

Table 3 demonstrates differences in anthropometric measures, metabolic factors, liver function, and SIIs by the severity of liver steatosis. By increasing the severity of liver steatosis, all of the anthropometric indices (except for HC) and metabolic parameters ( apart from TC and HDL-C) increased significantly (*p* < 0.05). Furthermore, liver function indices changed parallel with changes in NAFLD severity in terms of significant increases in ALT, AST, and ferritin as well as decreases in AST/ALT ratio. WBC (*p* < 0.001), monocytes (*p* = 0.035), and neutrophils (*p* < 0.001) also enhanced by the hepatosteatosis severity. Indeed, apart from PLR, all of the SIIs were significantly greater in grade II than in grade I liver steatosis (all *p* < 0.05) (Table 3).

**Table 3:** Participants' characteristics by the severity of steatosis.

	Mild (Grade 1) N = 140 Mean (SD)	Moderate (Grade 2) N = 98 Mean (SD)	*p*
Weight (Kg)	89.43 (16.22)	94.73 (15.69)	0.013*
Height (m)	1.67 (0.09)	1.68 (0.11)	0.382*
WC (cm)	104.73 (10.28)	109.49 (9.54)	< 0.001*
HC (cm)	113.67 (8.88)	115.55 (8.03)	0.096*
BMI (Kg/m^2^)	32.05 (4.87)	33.37 (3.72)	0.024*
WHR	0.92 (0.06)	0.95 (0.06)	0.001*
WHtR	0.63 (0.06)	0.65 (0.05)	0.002*
FBS (mg/dl)	92.71 (10.32)	95.91 (10.21)	0.019*
TG (mg/dl)	151.24 (70.40)	169.34 (86.60)	0.004*
TC (mg/dl)	173.37 (31.29)	186.67 (38.04)	0.078*
HDL-C (mg/dl)	44.72 (9.82)	44.07 (9.87)	0.618*
LDL-C (mg/dl)	126.99 (52.41)	150.83 (62.08)	0.002*
ALT (U/L)	26.96 (12.8)	37.24 (24.99)	< 0.001**
AST (U/L)	22.90 (7.03)	26.96 (12.90)	0.005*
AST/ALT	0.96 (0.40)	0.86 (0.38)	0.046*
Ferritin (ng/ml)	81.47 (73.84)	104.67 (76.92)	0.020**
WBC (*10^9^/L)	9.33 (2.20)	10.54 (1.85)	< 0.001*
Monocytes (*10^9^/L)	0.60 (0.60)	0.63 (0.25)	0.035**
Lymphocytes (*10^9^/L)	3.44 (0.78)	3.39 (0.67)	0.495*
Neutrophils (*10^9^/L)	5.27 (1.40)	5.91 (1.25)	< 0.001*
Platelets (*10^9^/L)	249.68 (66.33)	251.25 (49.93)	0.250*
MHR (*10^8^/mmol)	5.75 (6.61)	5.94 (2.71)	0.011**
MLR	0.18 (0.17)	0.19 (0.08)	0.039**
NLR	1.67 (0.89)	1.83 (0.63)	0.004**
PLR	77.60 (32.84)	77.92 (24.63)	0.222**

Mean ± standard deviation (SD) is presented for data.

Bold values indicate statistically significant *p* < 0.05.

*WC* waist circumference, *HC* hip circumference, *BMI* body mass index, *WHR* waist to hip ratio, *WHtR* waist to height ratio, *FBS* fasting blood sugar, *TG* triglycerides, *TC* total cholesterol, *HDL-C* high-density lipoprotein cholesterol, *LDL-C* low-density lipoprotein cholesterol, *ALT* alanine aminotransferase, *AST* aspartate aminotransferase, *AST/ALT* AST to ALT ratio, *WBC* white blood cell count, *MHR* monocyte to HDL-C ratio, *MLR* monocyte to lymphocyte ratio, *NLR* neutrophil to lymphocyte ratio, *PLR* platelets to lymphocyte ratio.

*p-value for Two-independent Sample t-test.

**p-value for Mann–Whitney U-test.

As Table 4 demonstrates, MHR was significantly associated with HDL-C (*p* < 0.001), LDL-C (*p* < 0.001), and AST/ALT (*p* = 0.019) ratio whereas metabolic factors including serum TG (*p* = 0.037), TC (*p* = 0.011), and LDL-C (*p* < 0.001) were significantly different among tertiles of MLR.

**Table 4:** Association of MHR and MLR with anthropometric indices, metabolic and liver function biomarkers.

	MHR (*10^8^/mmol)				MLR			
	≤ 4.02	4.03–6.19	> 6.19	P*	≤ 0.13	0.14–0.20	> 0.20	
BMI (Kg/m^2^)	32.13 (5.30)	33.18 (3.99)	32.71 (3.94)	0.341	32.37 (5.47)	32.95 (4.04)	32.46 (3.51)	0.698
WC (cm)	105.28 (10.16)	108.29 (9.55)	107.22 (10.51)	0.337	106.60 (10.88)	107.16 (10.00)	106.43 (9.85)	0.904
WHR	0.92 (0.06)	0.94 (0.05)	0.94 (0.06)	0.219	0.93 (0.06)	0.93 (0.06)	0.94 (0.07)	0.676
WHtR	0.64 (0.06)	0.65 (0.06)	0.63 (0.06)	0.389	0.64 (0.06)	0.64 (0.06)	0.63 (0.05)	0.571
FBS (mg/dl)	93.10 (10.55)	94.22 (9.89)	95.18 (10.65)	0.454	94.03 (11.62)	94.22 (9.50)	93.13 (9.84)	0.795
TG (mg/dl)	146.15 (69.09)	164.38 (76.63)	186.11 (87.87)	0.213	145.22 (74.76)	178.62 (67.35)	156.05 (74.78)	**0.037**
TC (mg/dl)	173.05 (36.48)	185.60 (33.40)	177.82 (34.63)	0.080	168.70 (33.88)	184.37 (31.30)	182.93 (37.70)	**0.011**
HDL-C (mg/dl)	48.54 (10.75)	45.58 (9.24)	39.62 (7.12)	** < 0.001**	44.00 (9.57)	44.70 (8.43)	43.97 (10.90)	0.879
LDL-C (mg/dl)	115.60 (52.87)	150.86 (60.31)	143.73 (54.82)	** < 0.001**	113.60 (50.58)	153.64 (55.49)	145.15 (60.02)	** < 0.001**
ALT (U/L)	27.47 (13.19)	32.15 (19.92)	34.53 (23.67)	0.071	28.97 (16.48)	29.60 (15.26)	36.12 (26.12)	0.058
AST (U/L)	24.21 (7.29)	23.65 (10.21)	25.53 (12.31)	0.495	24.74 (8.94)	23.27 (8.68)	25.71 (12.97)	0.368
AST/ALT	0.98 (0.33)	0.82 (0.23)	0.90 (0.41)	**0.019**	0.98 (0.39)	0.89 (0.43)	0.87 (0.38)	0.246
Ferritin (ng/ml)	83.33 (83.55)	92.97 (67.40)	99.09 (76.01)	0.426	78.84 (85.05)	87.62 (65.04)	102.40 (73.03)	0.160

Mean ± standard deviation (SD) is presented for data.

Bold values indicate statistically significant *p* < 0.05.

*MHR* monocyte to HDL-C ratio, *MLR* monocyte to lymphocyte ratio, *BMI* body mass index, *WC* waist circumference, *WHR* waist to hip ratio, *WHtR* waist to height ratio, *FBS* fasting blood sugar, *TG* triglyceride, *TC* total cholesterol, *HDL-C* high-density lipoprotein cholesterol, *LDL-C* low-density lipoprotein cholesterol, *ALT* alanine aminotransferase, *AST* aspartate aminotransferase, *AST/ALT* AST to ALT ratio.

*p-value for trend (Analysis of Variance (ANOVA)).

Table 5 shows the association of NLR and PLR with studied factors. Anthropometric indices including WC (*p* = 0.032), BMI (*p* = 0.047), and WHtR (*p* = 0.002) as well as FBS (*p* = 0.045), TG (*p* = 0.025), and LDL-C (*p* = 0.006) levels were significantly different in NLR teritles. Serum HDL-C (*p* = 0.036) and AST/ALT ratio (*p* = 0.001) were markedly related with PLR tertiles, while ALT levels were marginally associated with the tertiles of PLR (*p* = 0.051).

**Table 5:** Association of NLR and PLR with anthropometric indices, metabolic and liver function biomarkers.

	NLR				PLR			
	≤ 1.40	1.41–1.86	> 1.86	P*	≤ 64.69	64.70–84.50	> 84.50	P*
BMI (Kg/m^2^)	32.16 (5.33)	32.04 (3.94)	33.61 (3.81)	**0.047**	33.36 (5.33)	33.03 (4.12)	32.41 (3.85)	0.572
WC (cm)	106.49 (10.40)	104.69 (8.40)	108.88 (11.09)	**0.032**	107.57 (10.52)	107.39 (9.77)	105.07 (10.36)	0.230
WHR	0.93 (0.06)	0.93 (0.05)	0.94 (0.07)	0.575	0.94 (0.05)	0.94 (0.06)	0.93 (0.07)	0.548
WHtR	0.64(0.06)	0.62(0.06)	0.65(0.06)	**0.002**	0.63(0.06)	0.65(0.06)	0.63(0.06)	0.375
FBS (mg/dl)	93.55(9.89)	92.04(9.63)	96.06(10.91)	**0.045**	93.25(10.45)	94.96(9.09)	93.87(11.50)	0.579
TG (mg/dl)	141.03 (63.55)	175.20 (93.49)	158.07 (71.60)	**0.025**	160.68 (89.06)	151.22 (65.57)	160.96 (75.29)	0.666
TC (mg/dl)	172.05 (28.95)	180.49 (35.48)	182.66 (39.17)	0.142	177.96 (3.49)	172.59 (35.49)	185.28 (37.19)	0.069
HDL-C(mg/dl)	44.48 (10.60)	44.83 (7.83)	44.13 (10.92)	0.905	43.59 (10.05)	43.07 (8.30)	46.81 (10.78)	**0.036**
LDL-C (mg/dl)	118.99 (48.66)	148.92 (59.71)	145.23 (60.52)	**0.006**	135.15 (59.02)	141.41 (59.96)	134.38 (54.86)	0.708
ALT (U/L)	32.39 (20.49)	29.55 (15.04)	31.99 (22.82)	0.624	35.49 (20.76)	29.58 (17.43)	28.39 (19.84)	0.051
AST (U/L)	25.41 (11.43)	24.02 (8.48)	24.58 (10.53)	0.696	25.85 (11.41)	23.19 (9.57)	24.56 (9.25)	0.258
AST/ALT	0.90 (0.33)	0.92 (0.38)	0.94 (0.46)	0.819	0.82 (0.25)	0.90 (0.36)	1.04 (4.97)	**0.001**
Ferritin (ng/ml)	88.28 (90.33)	81.22 (60.37)	103.27 (74.92)	0.179	94.36 (91.37)	82.91 (62.28)	97.20 (71.00)	0.455

Mean ± standard deviation (SD) is presented for data.

Bold values indicate statistically significant *p* < 0.05.

*NLR* neutrophil to lymphocyte ratio, *PLR* platelets to lymphocyte ratio, *BMI* body mass index, *WC* waist circumference, *WHR* waist to hip ratio, *WHtR* waist to height ratio, *FBS* fasting blood sugar, *TG* triglyceride, *TC* total cholesterol, *HDL-C* high-density lipoprotein cholesterol, *LDL-C* low-density lipoprotein cholesterol, *ALT* alanine aminotransferase, *AST* aspartae aminotransferase, *AST/ALT* AST to ALT ratio.

*p-value for trend (Analysis of Variance (ANOVA)).

The correlations between SIIs and anthropometric indices, metabolic and liver function biomarkers are presented in Table 6. Only NLR was significantly correlated with BMI (r = 0.160, *p* = 0.013) while MLR and PLR were significantly correlated with liver function biomarkers, particularly in females and moderate steatosis. In addition, there were significant correlations between serum ferritin and all SIIs, apart from PLR. Within-gender associations between SIIs and the studied factors were tested (Data not presented in Tables), i.e. among females, LDL-C was significantly correlated with MHR (r = 0.266, *p* = 0.002), MLR (r = 0.191, *p* = 0.003), and NLR (r = 0.176, *p* = 0.045) whereas PLR was found to be correlated with serum ALT (r = -0.245, *p* = 0.004), AST/ALT ratio (r = 0.229, *p* = 0.008) and ferritin (r = 0.194, *p* = 0.025).

**Table 6:** Correlations of systemic inflammatory indices with anthropometric indices, metabolic and liver function biomarkers.

	MHR	MLR	NLR	PLR
BMI (Kg/m^2^)	0.001 (0.985)	0.032 (0.625)	0.160 **(0.013)**	0.004 (0.947)
WC (cm)	0.055 (0.404)	− 0.011 (0.869)	0.115 (0.076)	− 0.099 (0.127)
WHR	0.101 (0.121)	0.017 (0.792)	0.011 (0.864)	− 0.082 (0.207)
WHtR	− 0.051 (0.439)	− 0.024 (0.718)	0.117 (0.073)	0.005 (0.940)
FBS (mg/dl)	0.043 (0.513)	− 0.022 (0.737)	0.064 (0.325)	0.011 (0.865)
TC (mg/dl)	0.090 (0.168)	0.209 **(0.001)**	0.103 (0.114)	0.041 (0.526)
TG (mg/dl)	0.067 (0.308)	0.071 (0.279)	0.152 **(0.019)**	0.016 (0.801)
HDL-C (mg/dl)	− 0.395 **(< 0.001)**	0.012 (0.860)	− 0.009 (0.888)	0.137 **(0.036)**
LDL-C (mg/dl)	0.223 **(0.001)**	0.235 **(< 0.001)**	0.192 **(0.003)**	− 0.027 (0.678)
ALT (U/L)	0.154 **(0.018)**	0.044 (0.504)	− 0.032 (0.620)	− 0.212 **(0.001)**
AST (U/L)	0.016 (0.812)	− 0.016 (0.807)	0.007 (0.919)	− 0.018 (0.783)
AST/ALT	− 0.169 **(0.009)**	− 0.085 (0.191)	− 0.046 (0.481)	0.171 **(0.008)**
Ferritin (ng/ml)	0.159 **(0.015)**	0.178 **(0.006)**	0.157 **(0.015)**	0.123 (0.058)

Bold values indicate statistically significant *p* < 0.05.

*MHR* monocyte to HDL-C ratio, *MLR* monocyte to lymphocyte ratio, *NLR* neutrophil to lymphocyte ratio, *PLR* platelets to lymphocytes ratio, *BMI* body mass index, *WC* waist circumference, *WHR* waist to hip ratio, *WHtR* waist to height ratio, *FBS* fasting blood sugar, *TC* total cholesterol, *TG* triglyceride, *HDL-C* high-density lipoprotein cholesterol, *LDL-C* low-density lipoprotein cholesterol, *ALT* alanine aminotransferase, *AST* aspartae aminotransferase, *AST/ALT* AST to ALT ratio.

*r(p), *p*-value for Spearman correlation coefficient.

## Discussion

The present study investigated the association of a number of SIIs including MHR, MLR, NLR, and PLR with metabolic and anthropometric factors and also liver function in patients with NAFLD. The detection of chronic inflammation is considered a crucial goal for clinical specialists. Inflammatory biomarkers such as C-reactive protein and pro-inflammatory cytokines (e.g. interleukin 6 and tumor necrosis factor-α have been commonly studied to identify inflammatory states^17^. Furthermore, hepatosteatosis screening and monitoring are very important for the prevention of severe complications such as liver fibrosis and thereby finding reliable and simple markers of NAFLD in the blood seems to be necessary^3^. There is a growing recognition toward the critical role of systemic inflammation (intra- and extra-hepatic) assessment by relatively novel inflammatory indices such as NLR or MHR in NAFLD using a rapid, inexpensive, and routine blood test^2^.

Results of the present study demonstrated that apart from PLR, all of the SIIs significantly changed by increasing steatosis severity. Moreover, changes in NLR showed a significant association with anthropometric indices including WC, BMI, and WHtR, as well as levels of FBS, TG, and LDL-C. The findings also indicate the relations between lipid profile and all studied SIIs, notably MHR and MLR. All of the SIIs exhibited associations with some liver function indices as well. MHR was positively correlated with the metabolic risk factors of NAFLD while, oppositely, PLR was considered as a preventive marker of NAFLD. Our results also showed that anthropometric measures, liver function biomarkers, MHR, and MLR were significantly different between the genders. There is cumulative evidence indicating that inflammatory conditions more frequently occur in men whereas chronic inflammatory diseases with lower mortality rates mostly affect women^18–20^.

We documented significant increases in SIIs (in terms of MHR, MLR, and NLR) parallel to the changes of all anthropometric and liver function indices as well as lipidemia, by an increasing trend in hepato-steatosis severity. In this context, the association between inflammation, SIIs, and dyslipidemia has been well established in many conditions such as rheumatoid arthritis, neurological disease, depression, and mood disorders^20–23^. For example, Gong et al.^24^ in their study on patients with ischemic stroke showed that SIIs including NLR, PLR, and lymphocyte-monocyte ratio (LMR) were greater in those with neurological problems than controls. It is suggested that high PLR values could be used for the prediction of thrombosis and inflammatory disturbances as well as neurological outcomes in patients with acute ischemic stroke^24^. Additionally, the effectiveness of PLR in the prediction of all-cause and cardiovascular mortality in the maintenance of patients undergoing hemodialysis is also documented^25^. Moreover, SIIs have been implicated in the assessment of endocrine, metabolic, and CVDs, as well as cancers^2,6,26^.

Despite the relatively weak association found between anthropometric indices (BMI, WC, and WHtR) and serum FBS levels with changes in NLR values in this study, other SIIs failed to present any relation to those factors. The findings also indicate the relations between lipid profile and all studied SIIs, notably MHR and MLR. Although these associations are not well established by previous studies on obesity-related conditions such as Mets and NAFLD yet, patients with Mets have shown significantly higher MHR, NLR, and PLR whereas lower LMR compared with healthy subjects^10,27–30^. However, it should be noted that gender, BMI, and fasting hyperglycemia could influence SIIs and thereby, lead to variations in findings^26^. Our data shed light on the use of NLR, MHR, and MLR for the assessment of metabolic outcomes of NAFLD; namely by the hypothesis on the ability of MHR and MLR in the assessment of lipidemia as well as the application of NLR in obesity and glycemia assessment. Based on current evidence, MHR has been suggested for showing the complex interplay between pro-inflammatory/pro-atherogenic factors as well as lipid profile^26^. Therefore, it appears that high MHR might be a useful marker for the evaluation of lipidemia in NAFLD and other metabolic diseases.

Regarding the possible application of NLR for the assessment of glycemia, in line with our results, several studies have reported the relationship between higher NLR and poor glycemic control, macro- and micro-vascular diabetic complications in T2DM patients^31,32^. It appears that NLR could be used as a predictive marker of glycemic status in metabolic disorders.

As NAFLD is the hepatic manifestation of metabolic dysregulations, it involves a complex interplay among factors including excessive body fat deposition, impaired glucose and lipid homeostasis, oxidative stress, and chronic inflammation^33^. The progression of pro-inflammatory signaling pathways leads to impairments in liver function characterized by hepatic histologic findings^34^. To date, limited studies have investigated the relationship between SIIs and NAFLD^2,12,35,36^. In the present study, all of the SIIs showed correlations with a number of liver function biomarkers. These findings suggest increased MHR and PLR values as the progressive and preventive markers of NAFLD, respectively. A recent study reported the relationship between NLR and PLR with NAFLD, after adjusting for potential confounders^12^. Similar to our findings, Zhao et al.^2^ reported that MHR was significantly higher in patients with NAFLD than in healthy controls and suggested that MHR is strongly associated with the risk of NAFLD compared with NLR, PLR, and LMR. Indeed, Huang et al.^37^ reported that MHR was significantly correlated with the high risk of NAFLD, even after adjusting for age, sex, BMI, WC, ALT, TG, TC, FBS, and blood pressure. MHR is calculated based on monocytes, which are considered an important component of the immune system and a vital mediator of both chronic and acute inflammation, and thereby, induces immune-inflammatory responses^38^. In addition, HDL-C by producing an anti-inflammatory response to macrophages and mediating cholesterol consumption could be necessary for the prevention of inflammatory diseases^39^. Therefore, increased MHR is associated with diabetic nephropathy, predicting Mets and NAFLD^10,40^.

Regarding PLR, several studies similarly suggested that PLR may be more useful as a marker in determining the increased thrombotic state and inflammatory response in morbid obesity, obstructive sleep apnea–hypopnea syndrome, overall survival in patients with NAFLD-related hepatocellular carcinoma^5,36,41,42^. Taken together, it seems that PLR has relatively higher diagnostic accuracy for liver fibrosis^35^. It appears that the combination of NLR and MLR (as the markers of anthropometric and lipid-related changes) with PLR and MHR (as the indicators of liver function and metabolic regulation) could present a comprehensive perspective of NAFLD.

Accordingly, these findings could be explained by the potent role of inflammation, in terms of peripheral blood leukocytes in the progression of NAFLD^43^. Stimulated hepatocyte neutrophil infiltration is followed by macrophage recruitment and cellular damage, which activates pro-inflammatory pathways as well as oxidative stress^43^. In addition, liver stellate cells facilitate the production of hepatic reactive oxygen species and the progression of liver fibrosis through increasing neutrophils survival. Current evidence also suggests an interplay between platelets and pro-inflammatory cells, which is followed by increased chemokine and immune-inflammatory mediator secretion, which subsequently induce NAFLD^43^. Taken together, changes in the balance and the total count of neutrophils, lymphocytes, and platelets in the blood could reflect a systemic inflammatory state and reflect the risk of NAFLD and its related outcomes^43^.

Some limitations should be acknowledged when interpreting the findings of this study. First, the nature of cross-sectional cannot make a judgment regarding the causal relationship between SIIs and NAFLD. Larger sample size with various spectrum of NAFLD severity in different age groups is needed to confirm these results. Furthermore, sarcopenia, which is a progressive and systemic skeletal muscle disease, has attracted considerable attention as one of the pathological conditions involved in the development and progression of NAFLD. Sarcopenia has been reported to be closely associated with NAFLD. It causes sarcopenia-related obesity, which increases the risk of nonalcoholic steatohepatitis, liver fibrosis, and CVD^44–46^. However, due to the lack of data on sarcopenia status in our patients, we were unable to explore the association of SIIs with sarcopenia in the current study, which is suggested to be evaluated in future studies. The current study has several strengths too. Subgroup comparison based on NAFLD severity, and considering not only metabolic but also anthropometric indices and the biomarkers of liver function in relation to SIIs are considered as the strengths of the present study.

In conclusion, SIIs, which can be easily measured in clinical practice, are related to the presence and severity of NAFLD. Moreover, we suggested that PLR and MHR by themselves or combined with other SIIs could be used in NAFLD monitoring. However, further research is required to confirm these findings.

## Data Availability

The datasets used and/or analyzed during the current study are available from the corresponding author on reasonable request.

## Abbreviations

ALT: Alanine aminotransferase
ANOVA: Analysis of Variance
AST: Aspartate aminotransferase
AST/ALT: Aspartate aminotransferase to alanine aminotransferase ratio
BMI: Body mass index
CBC: Complete blood count
FBS: Fasting blood sugar
HC: Hip circumference
HDL-C: High-density lipoprotein cholesterol
LDL-C: Low-density lipoprotein cholesterol
LMR: Lymphocyte to monocytes ratio
Mets: Metabolic syndrome
MHR: Monocytes to High-density lipoprotein cholesterol ratio
MLR: Monocytes to lymphocyte ratio
NAFLD: Non-alcoholic fatty liver disease
NLR: Neutrophil to lymphocyte ratio
PLR: Platelet to lymphocyte ratio
SIIs: Systemic inflammatory indices
T2DM: Type 2 diabetes mellitus
TC: Total cholesterol
TG: Triglycerides
WC: Waist circumference
WHR: Waist to hip ratio
WHtR: Waist to height ratio
